# Out-of-Pocket Costs Attributable to Dementia: A Longitudinal Analysis

**DOI:** 10.1093/geroni/igaa057.1539

**Published:** 2020-12-16

**Authors:** Melissa Oney, Lindsay White, Norma Coe

**Affiliations:** 1 University of Pennsylvania, PHILADELPHIA, Pennsylvania, United States; 2 RTI International, Seattle, Washington, United States; 3 University of Pennsylvania, Philadelphia, Pennsylvania, United States

## Abstract

Alzheimer’s disease and related dementias (ADRD) affects 5.5 million Americans, and is expensive despite the lack of a cure or even treatments effective in managing the disease. The literature thus far has tended to focus on the costs to Medicare, despite the fact that one of the main characteristics of ADRD (the loss of independence and ability to care for oneself) incurs costs not covered by Medicare. In this paper, we use survey data for 2002-2014 from the Health and Retirement Study to estimate the out-of-pocket costs of ADRD for the patient and their family through the first 8 years after onset of symptoms, as defined by a standardized 27-point scale of cognitive ability. A two-part model developed by Basu and Manning (2010) allows us to separate the costs attributable to ADRD into two components, one driven by differences in longevity and one driven by differences in utilization. We consider total out-of-pocket expenditures, as well as out-of-pocket expenditures by category (i.e. hospital, nursing home, doctor, prescription drug, and other). Our results suggest that the out-of-pocket costs of ADRD are quite substantial over the first 8 years after onset. We also find that out-of-pocket spending is decreasing over the first 8 years, similar to the trend seen in Medicare expenditures. The results of this study highlight the financial burden of ADRD, particularly for the population paying out-of-pocket for care.

